# Fibronectin Affects Transient *MMP2* Gene Expression through DNA Demethylation Changes in Non-Invasive Breast Cancer Cell Lines

**DOI:** 10.1371/journal.pone.0105806

**Published:** 2014-09-10

**Authors:** Isabela T. Pereira, Edneia A. S. Ramos, Erico T. Costa, Anamaria A. Camargo, Graciele C. M. Manica, Liliane M. B. Klassen, Andressa Chequin, Karin Braun-Prado, Fábio de O. Pedrosa, Emanuel M. Souza, Fabricio F. Costa, Giseli Klassen

**Affiliations:** 1 Department of Basic Pathology, Federal University of Parana, Curitiba, Paraná, Brazil; 2 Ludwig Institute for Cancer Research (LICR) at Molecular Oncology Center, Sirio-Libanes Hospital, São Paulo, São Paulo, Brazil; 3 Department of Biochemistry and Molecular Biology, Federal University of Parana, Curitiba, Parana, Brazil; 4 Cancer Biology and Epigenomics Program, Ann and Robert Lurie Children’s Hospital of Chicago Research Center and Department of Pediatrics, Northwestern University’s Feinberg School of Medicine, Chicago, Illinois, United States of America; Florida State University, United States of America

## Abstract

Metastasis accounts for more than 90% of cancer deaths. Cells from primary solid tumors may invade adjacent tissues and migrate to distant sites where they establish new colonies. The tumor microenvironment is now recognized as an important participant in the signaling that induces cancer cell migration. An essential process for metastasis is extracellular matrix (ECM) degradation by metalloproteases (MMPs), which allows tumor cells to invade local tissues and to reach blood vessels. The members of this protein family include gelatinase A, or MMP-2, which is responsible for the degradation of type IV collagen, the most abundant component of the basal membrane, that separates epithelial cells in the stroma. It is known that fibronectin is capable of promoting the expression of MMP-2 in MCF7 breast cancer cells in culture. In addition, it was already shown that the *MMP2* gene expression is regulated by epigenetic mechanisms. In this work, we showed that fibronectin was able to induce *MMP2* expression by 30% decrease in its promoter methylation. In addition, a histone marker for an open chromatin conformation was significantly increased. These results indicate a new role for fibronectin in the communication between cancer cells and the ECM, promoting epigenetic modifications.

## Introduction

Breast cancer is the most prevalent cancer in women both in the developed and in the developing world. In the United States, it is estimated that breast cancer is the leading cause of all cancers (29%) and the second leading cause of death (14%) [1]. In Brazil, 57,120 new cases of breast cancer were estimated for 2014 [2].

From a clinical perspective, metastasis is considered one of the most important stages of tumor progression because it accounts for more than 90% of cancer deaths [3], [4], [5]. At some point during the development of most human cancers, cells from the primary tumor may invade adjacent tissues and migrate to distant sites, establishing new colonies called metastasis [5]. The tumor microenvironment is now recognized as an important participant in tumor progression, spreading and in treatment response. An essential process in the establishment of metastasis is the extracellular matrix degradation, which allows tumor cells to invade local tissues, leave the primary tumor and reach blood vessels [5]. This process is primarily influenced by the activity of proteases that are released at the site of the tumor, including the group of matrix metalloproteases (MMPs) [6].

Among the members of this family of proteins family, gelatinase A (MMP-2) is implicated in local tumor invasion and metastasis [7]. This process primarily occurs through the degradation of type IV collagen, the most abundant component of the basal membrane; this degradation is involved in the process of epithelial cells separation from the stroma [6], [7].

It is known that in culture, fibronectin is capable of promoting the expression of MMP-2 in fibrosarcoma cells [8], cervical cancer cells [9], MCF7 breast cancer cells [10] and prostate cancer cells [11]. In addition, it has already been shown that *MMP2* gene expression is regulated by epigenetic mechanisms [12], [13]. Therefore, the aim of this study was to evaluate the effects on the DNA methylation of the *MMP2* promoter after inducing *MMP2* expression with fibronectin. In order to achieve this goal, we used the well-characterized MCF7 and MDA-MB-436 breast cancer cell lines in our study to obtain more insights into the role of fibronectin in cancer metastasis.

## Results

### *MMP2* gene activation by both 5-aza-2′-deoxycytidine and fibronectin in MCF7 cells

**Figure 1:**
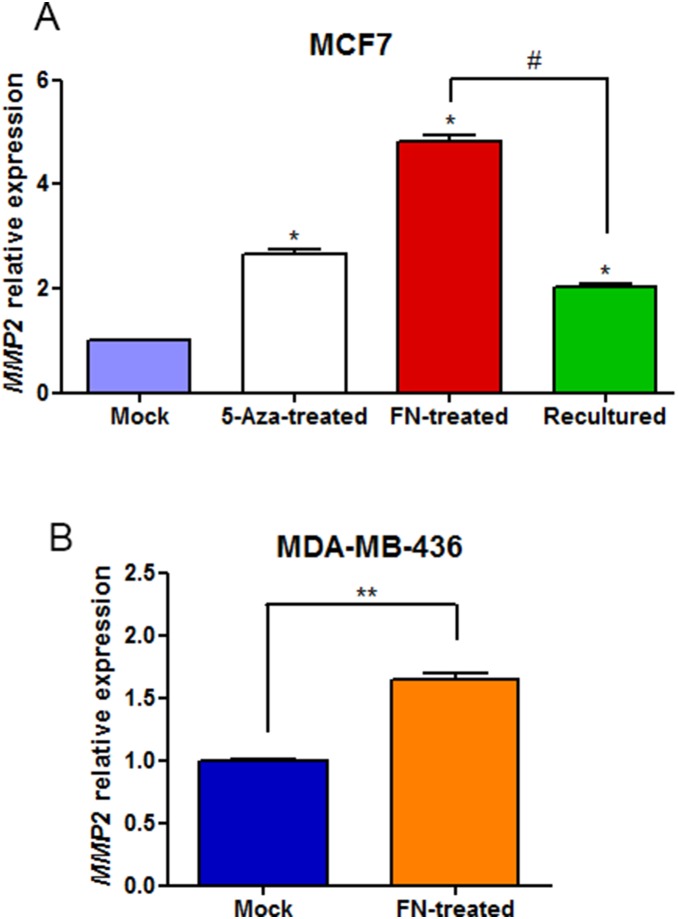
*MMP2* expression after treatment in breast cancer cell lines. A: The expression of *MMP2* after treatments in MCF7 cells was shown. Mock (blue); 5-Aza-treated (white), FN-treated (red) and recultured (green). Results were expressed as mean S.E.M. and statistical comparison was performed using *t test* analysis. *p<0.05 when compared to mock; #*p*<0.05 when FN-treated compared to recultured. B: The expression of *MMP2* in MDA-MB-436 cells, mock and after FN treatment were shown. Mock (blue) and FN-treated (orange) were expressed as mean S.E.M. and statistical comparison was performed using Student’s *t* Test. **p<0.05 when compared to mock.

MCF7 breast tumor cells were submitted to four experiments: in serum free medium (SFM) without fibronectin (mock); treated with 5-aza-2′-deoxycytidine (5-Aza-treated) for seven days; in SFM containing fibronectin (FN) for five hours (FN-treated); and FN-treated cells were transferred to new culture dishes and maintained in SFM in culture for additional 48 hours without fibronectin (recultured cells). After these treatments, *MMP2* expression was assessed by qRT-PCR (Fig. 1). The MCF7 5-Aza-treated showed a 2.6-fold increase in *MMP2* expression compared to the mock. In contrast, 5 hours of the FN treatment induced in 5-fold the expression of *MMP2* (Fig. 1A). In addition, 48h after the FN withdrawal recultured cells showed decreased *MMP2* expression (2-fold compared to FN-treated) (Fig. 1A).

In order to confirm that these results were not restricted to the MCF7 tumor cell line, we analyzed the effects of FN treatment in *MMP2* expression in the breast cancer cell line MDA-MB-436. Accordingly, *MMP2* expression in FN-treated MDA-MB-436 cells was 1.6-fold significantly higher than mock (Fig. 1B).

### MMP-2 enzymatic assay by zymography

Under these experimental treatments, MMP-2 activity in MCF7 cells was measured by zymography assays. MMP-2 enzyme isoforms of 72 kDa (pro-MMP-2) and 62 kDa (active MMP-2) were clearly observed after 5-Aza and FN treatments compared with conditioned SFM from mock cells that did not digest the gelatin substrate (Fig. S1). These results corroborate the gene expression data and indicate that MMP-2 is secreted as an active proteinase after FN treatment in MCF7 cells (Fig. S1).

### Fibronectin and changes in the global methylation profile of the *MMP2* gene promoter

**Figure 2:**
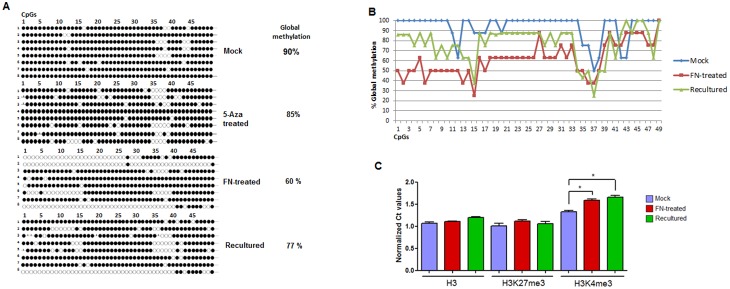
Epigenetic changes in the *MMP2* gene promoter in MCF7 cells. A: Sequencing of the *MMP2* gene promoter. Closed and open circles represent methylated or unmethylated CpGs, respectively. On the left the number represent the sequenced clones. The 49 analyzed CpGs in the *MMP2* promoter region are shown. Mock cells are at the top, above is 5-Aza-treated, hereafter FN-treated, recultured (MCF7 FN-treated and then cultured for 48 hours without fibronectin). The global methylation percentage is also shown at the right. B: Graphical analysis of CpG methylation pattern in the *MMP2* promoter gene. The percentages of CpGs that were methylated in MCF7 mock (blue), MCF7 FN-treated (red) and MCF7 recultured (green) were shown. C: ChIP quantitative PCR analysis. Ct values were normalized between target and endogenous control (*MMP2*/*GAPDH*) and the results of mock (blue), FN-treated (red) and recultured (green) cells are shown. At the bottom the samples are separated according to the antibody used in the immunoprecipitation. Statistical comparison was performed using Student’s *t* Test. *p<0.05.

To assess whether fibronectin has the potential to change the methylation profile in the promoter region of the *MMP2* gene, the DNA from MCF7 mock, 5-Aza-treated and FN-treated cells was extracted and subjected to sodium bisulfite conversion. The promoter region of *MMP2* was amplified, purified, cloned and sequenced (Fig. 2A) to evaluate epigenetic changes.

The sequence analysis showed that the *MMP2* gene promoter had 90%, 85% and 60% of global DNA methylation in MCF7 mock, 5-Aza-treated and FN-treated cells, respectively (Fig. 2A). In addition, to exclude cell lineage specificity, we analyzed the promoter *MMP2* sequence in the MDA-MB-436 mock and FN-treated cells. The similar demethylation effect was observed with 90% and 52% of global methylation in mock and FN-treated cells, respectively (Fig. S2).

To assess whether *MMP2* promoter demethylation would be maintained upon FN withdrawal, we performed a re-culture experiment in the absence of fibronectin. In this condition, MCF7 recultured cells showed 77% of methylation (Fig. 2A). The sequence data was represented in a graph and the CpG methylation status showed a clear difference between these three experimental conditions, particularly the CpGs 7–12 and 18–26 that were 40–50% demethylated in FN-treated and partially remethylated in MCF7 recultured cells (Fig. 2B).

### Effects of fibronectin on the migration or invasion of MCF7 cells

**Figure 3:**
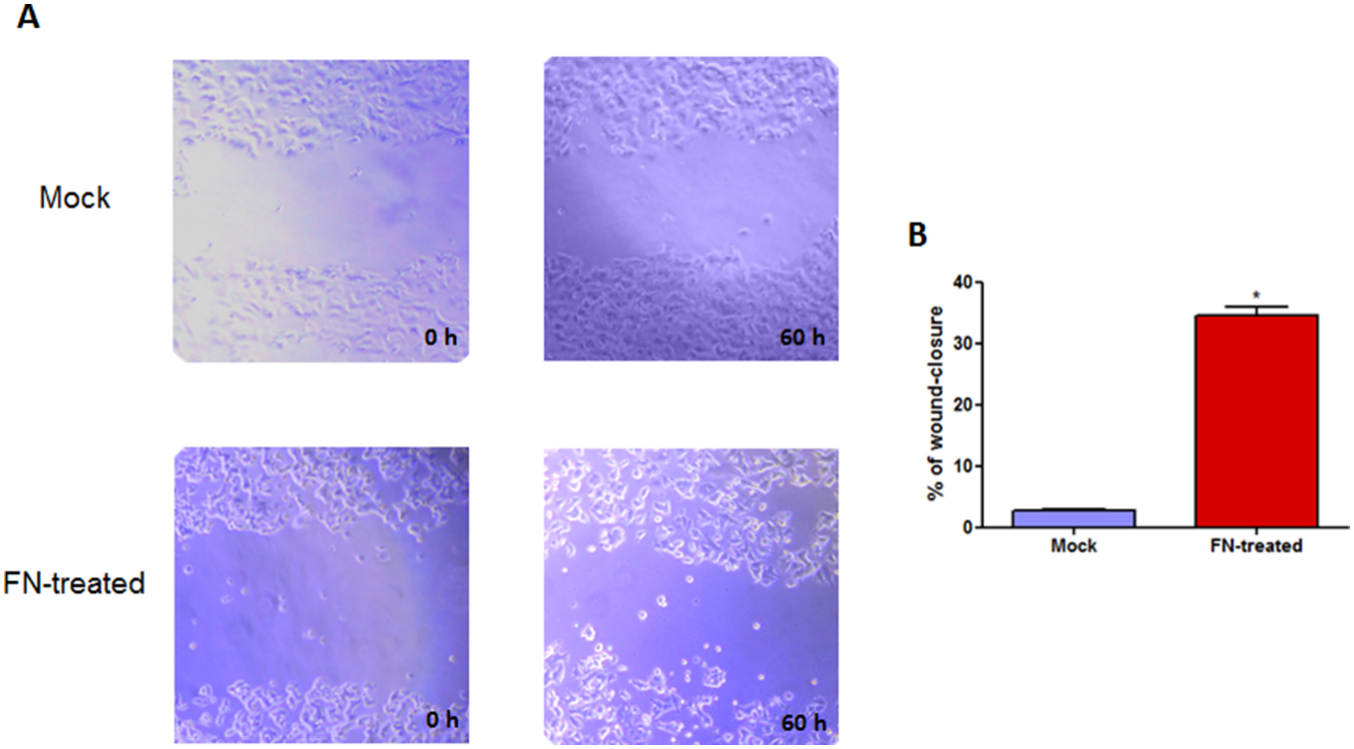
Wound healing assay in MCF7 cells. A**:** Representative images from mock and FN-treated cells were shown. The scratched cells in a line had images obtained under un inverted optical microscopy (20X). B**:** The graphic represents % of wound-closure after 60 h in culture. Statistical comparison was performed using Student’s *t* Test. *p<0.05.

Given the increased expression of *MMP2* after incubation with fibronectin (FN), the migratory potential of FN-treated cells was tested by wound-healing and transwell migration assays. Microscopic examination at 60 h revealed a statistically significant increase in the wound-closure rate of FN-treated MCF7 cells compared with mock cells (Fig. 3). In contrast, using FN or collagen type-I as haptotatic factors in transwell assays, were not observed significant differences in the migratory rates between mock and FN-treated MCF7 cells (Fig. S3).

Complementary, the invasive ability of MCF7 cells was evaluated in a spheroid three-dimensional culture system (3D) after mock or FN treatments. 3D cultures of MCF7 cells showed that FN-treated cells did not acquire significant invasive potential despite of increased levels of active *MMP2* (Fig. S4). These results indicate that despite of increased secretion of active MMP-2 (Fig. S2) and increased migratory ability observed in wound healing assays (Fig. 3), FN treatments were not sufficient to activate a fully invasive program in MCF7 cells under these experimental conditions.

### Chromatin Immunoprecipitation (ChIP) analysis

To assess histone modifications associated with the *MMP2* promoter gene, MCF7 mock, FN-treated and recultured cells were chromatin immunoprecipitated with antibodies associated with inactive chromatin (H3K27me3) [14] or with active chromatin (H3K4me3) [14]. The ChIP qPCR was performed for the *MMP2* promoter region as described [12]. The results of these experiments are shown (Fig. 2C).

The endogenous control H3 remained similar between the mock, FN-treated and recultured cells as expected. The transcription-silencing marker H3K27me3 remained similar in all conditions (Fig. 2C). In contrast, the H3K4me3 histone modification representing open chromatin was slightly but significantly increased in the *MMP2* promoter region after fibronectin treatment and recultured cells (Fig. 2C).

## Discussion

The accumulation of genetic and epigenetic changes is commonly believed to promote cancer development. This concept provides the basis for our knowledge of cancer progression, but it cannot explain the heterogeneity in tumor cell growth, invasion and resistance to therapy. Approximately 90% of deaths from breast cancer are due to metastasis [3], [4]. Metastasis is a complex and multistep process that requires the coordination of a number of properties by tumor cells. These properties include altered cell-cell and cell-substratum adhesion, increased motility, altered growth control, the ability to produce angiogenic factors, and increased expression of proteases [15]. Different groups of genes that can be activated or silenced in the metastatic process include the metalloproteases, especially the *MMP2* gene [6]. In addition to intrinsic factors of tumor cells, the extracellular matrix (ECM) is a key component of a cell’s microenvironment that cooperates with extracellular molecules to relay signals into cells. The stromal ECM protein fibronectin is essentially absent from normal adult breast tissue [16] and increased fibronectin mRNA and protein levels have been detected in breast tumors stroma [17], [18]. It is already known that fibronectin is capable of promoting the expression of MMP-2 in breast tumor cell lines [10], [19]. In contrast, it has been shown that this gene is regulated by epigenetic changes, including DNA methylation and histone modifications [12], [13]. Similarly to an epigenetic repression, sequencing the *MMP2* promoter of MCF7 mock cells revealed 90% of methylation. 5-aza and FN-treated MCF7 cells showed a 2.6 and 5-fold increase, respectively, in *MMP2* gene expression compared to mock cells (Fig. 1A). In agreement, zymography assays showed that the MMP-2 proteinase was active and secreted by MCF7 cells after inductive treatments (i.e. 5-Aza and FN, Figure S1). In 5-Aza-treated cells, despite the expression of the *MMP2* gene, a discrete demethylation in the promoter region was observed (Fig. 2A). Another study showed a similar result, and the authors noted that the inhibition of DNMT1 by 5-Aza treatment affected the expression of *MMP2* indirectly through methylation-independent mechanisms [13].

In contrast, FN-treated MCF7 cells showed a 5-fold increase in *MMP2* gene expression and 60% of global methylation (Fig. 2A). To exclude the possibility that these results are restricted to MCF7 tumor cells, we analyzed the *MMP2* promoter sequence in the MDA-MB-436 breast cancer cell line. The similar demethylation effect was observed with 90% and 52% of global methylation in mock and FN-treated MDA-MB-436 cells, respectively (Fig. S2). This is the first time that DNA demethylation was observed in the *MMP2* gene induced by a protein from extracellular matrix (ECM). The ECM or the microenvironment in which the cells are embedded is composed of a network of fibrilar molecules, including collagen and elastin; glycoproteins, such as laminin and fibronectin; and proteoglycans [16]. The epithelial-mesenchymal transition (EMT) is a phenotypic conversion that occurs during embryonic development and tissue remodeling, and it is essential for the development of metastasis [20]. EMT is a dynamic and reversible process that can occur in cells located at the tumor periphery. This process is triggered by signals, such as TGF, TNF and Wnt [21], [22], received from the microenvironment, and it has recently been proposed that fibronectin has an essential role in the establishment of EMT in breast cancer cells [23]. Thus, the results of the demethylation of the *MMP2* gene promoter after culturing with fibronectin could represent a very important step for metastasis. The epigenetic event seems to be a key regulatory mechanism in the metastasis process [24] and the *MMP2* gene promoter appears to be a suitable target for DNA demethylation following fibronectin signaling.

MCF7 cells have a low migration rate and weak invasive activity [25]. Recently, ectopic overexpression of *MMP2* in MCF7 cells was shown to result in enhanced motility and invasiveness in breast [26], ovarian [27] and lung cancer cells [28]. In the present study, we found a statistically significant increase in cell migration of FN-treated MCF7 cells compared with mock cells in wound healing assays (Fig. 3). This result highlights a functional role of the FN-induced MMP-2 proteinase in MCF7, a non-invasive breast tumor cell line, connecting the *MMP2* promoter demethylation with a phenotypical change in tumor cells. In contrast, in the haptotaxis migration assays, FN-treated cells did not change the migratory behavior toward FN or collagen type-I compared with mock cells (Fig. S3). One possible explanation to this apparent contradiction are the differences in cell preparation. While haptotaxis migration assays mandatorily require cell suspension and disruptions in the cell–cell and cell–ECM interactions, in wound healing assays cells migrate as a collective sheet in close contact with the substrate [29]. Moreover, an invasive assay mimicking the tumor three-dimensional architecture, known as MTS (multicellular tumor spheroids), was performed. However, no invasive phenotype was observed (Fig. S4), indicating that although FN promotes activation and secretion of MMP-2 by non-invasive MCF7 cells, the proposed treatment was not sufficient to make MCF7 fully invasive. We believe that other factors and players may have a combined role in this process.

The next step was to evaluate if the methylation pattern observed in FN-treated cells was transient or stable. We recultured the FN-treated cells for 48h in the absence of fibronectin and the *MMP2* expression significantly declined in this condition (Fig. 1A). Interestingly, sequencing of the *MMP2* promoter revealed a global methylation of 77% (Fig. 2A), showing that the demethylation pattern observed in the FN-treated cells was transient. These results support the hypothesis that demethylation is a dynamic, rapid and transient process and that the gene was likely re-methylated when the stimulus was removed [30], [31].

It has become increasingly clear that DNA methylation and histone lysine methylation systems are highly interrelated [32], [33]. In order to study how this connection could be involved in the *MMP2* promoter in MCF7 cells, we performed the chromatin immunoprecipitation assays (ChIP) with MCF7 mock, FN-treated and recultured cells. The H3K27me3 was similar in all tested conditions, however the H3K4me3 open chromatin mark was significantly higher in the FN-treated condition and recultured cells (Fig. 2C). Recently, reviews discussed that H3K4me3 is associated with non-methylated DNA state in active gene promoters [32], [33]. In contrast, the H3K27me3 is associated with repression of transcription and it seems to promote chromatin compaction [33]. Apparently, the H3K27me3 does not directly impair transcriptional reactivation but may be involved in providing imprints to facilitate subsequent gene silencing if the activating signal decays [34]. Many promoters in embryonic stem (ES) cells harbor a distinctive histone modification signature that combines the activating histone H3K4me3 mark and the repressive H3K27me3 mark. These bivalent domains are considered to poise expression of developmental genes, allowing timely activation while maintaining repression in the absence of differentiation signals [35]. The presence of both histone markers and DNA methylation patterns were recently studied in epithelial-mesenchymal transition (EMT) in Twist1 induced mammary epithelial cells [36]. Overall, the study showed that the number of genes marked by H3K4me3 and also by both H3K4me3 and H3K27me3 (bivalent) was increased following Twist1-induced EMT [36]. Besides, when DNA-hypermethylated genes were demethylated and reexpressed they adopted a bivalent chromatin pattern in colon cancer cells [37]. These reports can help explain the observed bivalent markers in the *MMP2* promoter FN-treated MCF7 cells.

Another important result was the re-methylation process observed in the recultured cells (Fig. 2A e 2B). A similar result was verified in colorectal carcinoma cells treated with 5-Aza [30]. The treated cells showed DNA demethylation of some genes and their reactivation, but the treatment was not able to resolve the bivalent chromatin. Interestingly, upon drug withdrawal, a re-methylation was reached [30]. Thus, it can be suggested that the presence of H3K27me3 can explain the rapid DNA re-methylation of *MMP2* in the absence of fibronectin. However, additional antibodies for other open and closed histone markers will be necessary to strengthen this hypothesis.

We propose that histone marks assist in the protection of gene methylation to ensure *MMP2* gene inactivation; however, these marks were not involved in the demethylation process. Thus, it appears that the dynamics of the DNA demethylation of the *MMP2* promoter region and the subsequent expression of the gene is an active and fast process. We provide the first evidence that a molecule -fibronectin- present in the tumor microenvironment is capable of inducing DNA demethylation and initiating changes in histone marks to activate an essential gene associated with metastasis.

## Materials and Methods

### Cell culture

The MCF7 and MDA-MB-435 and MDA-MB-436 cell lines were obtained from ATCC. Cells were cultured in RPMI 1640 medium (Gibco) supplemented with 10% fetal bovine serum (FBS) (Gibco), 2 mM glutamine and 40 mg/mL garamycin, following the protocol suggested by ATCC. For fibronectin treatment the cells were washed twice with PBS, trypsinized, centrifuged, and washed three times with RPMI without fetal bovine serum (FBS). After the washes, the cells were resuspended in RPMI without FBS and counted. Approximately 3×10^5^ MCF7 cells were treated with fibronectin [10] (25 µg/mL, Sigma Aldrich) or with serum-free medium (SFM) without fibronectin (mock) for 5 hours. The plates were incubated in a 37°C incubator with a 5% CO_2_ atmosphere.

### 5-Aza-2′-deoxycytidine treatment

Approximately 3×10^5^ MCF7 cells were treated with 1 µM 5-aza-2′-deoxycytidine (5-Aza-treated) (Sigma Aldrich) [38]. The medium was changed every day for seven days, and no significant cell death was observed.

### Quantitative Real-Time PCR for *MMP2* gene expression

RNA was extracted using the AllPrep kit (Qiagen) following the manufacturer’s instructions. Complementary DNA was prepared from 500 ng RNA in a 20 µL reaction volume containing 0.5 mM deoxyribonucleotide triphosphate, 1 µM oligodT, 10 U RNAsin (Promega) and Sensiscript reverse transcriptase with buffer (Qiagen). The negative control consisted of adding all the products needed for cDNA synthesis except the Sensiscript reverse transcriptase.

*MMP2* gene was quantified by qPCR. The internal controls consisted of sequences for the genes *GAPDH* and *HPRT* (600 nM) [39]. For this assay, we performed an efficiency curve for each primer pair and sample diluent. The cell line MDA-MB-435, which expresses the *MMP2* gene, was used as a positive control. Real-Time PCR was performed with 1x SYBR Green (Applied Biosystems) on the StepOne Plus equipment (Applied Biosystems).

### Metalloprotease activity by Zymography

The MMP-2 metalloproteinase activity was assessed using the zymography technique [40]. To evaluate MMP-2 activity, we used an 8% SDS-PAGE gel co-polymerized with 0.1% gelatin (Biorad). The conditioned serum-free medium (SFM) was concentrated with Sepharose-Gelatin resin (Sigma Aldrich) [10] and mixed with the electrophoresis buffer without reducing agents. After electrophoresis, the gel was washed in 2.5% Triton-X to completely remove SDS. Then, the gel was placed in incubation buffer (5 mM CaCl_2_ and 1 mM ZnSO_4_) at 37°C for approximately 48 hours. Discolored bands in the gel were visualized to quantify gelatinase activity [41].

### Wound healing Assay

MCF7 mock or FN-treated cells (200,000 cells) were cultured in confluent monolayers in 24 well plates. The cells were serum depleted for 4 hours and the FN-treatment was done as described. The monolayers were wounded as a line across the well with the use of a 200 µl pipette tip was made, then washed twice with serum free media to remove cell debris and incubated in a 37°C incubator with a 5% CO_2_ atmosphere. The cell free wound area was photographed at the indicated times with a digital camera connected to an inverted microscope. Images were analyzed by Image J software. Wound healing was calculated as the proportion of migrating cells compared with the initial wound area [29]. The results were obtained from three independent triplicate assays.

### Transwell migration assay

The transwell plates (Costar) containing 8 µm membranes were used for this assay. The lower chambers were pre-incubated with a solution of 20 µg/mL type I collagen (BD Biosciences) in PBS (300 µL/well). Before testing, the wells were washed twice with PBS and then maintained in RPMI without FBS at 4°C. After the treatments, cells were released from the plate with trypsin, and 4×10^4^ cells in 100 µL of RPMI (no FBS) were placed in the upper chamber of the transmigration well. The plate was then incubated in 5% CO_2_ at 37°C for 14 hours. This technique was performed in duplicates and on two different days as a control for our treatment. The quantification of migratory cells was performed with DAPI (4′, 6-diamino-2-phenylindole). After the migration (14 hours) the transwells were washed with 1x PBS, and the cells were fixed with 1% formaldehyde for 10 minutes at room temperature. After washing with PBS, the top chamber of each well was dried with the aid of a cotton swab. The cell nuclei were stained with DAPI (1∶1000) for 5 minutes and washed 3 times in PBS. The number of cells in 20 optical fields independent of each experimental condition was evaluated in a fluorescence microscope (UV 330–338).

### 3D Invasion assay

Forty-eight-well plates were previously prepared with low melting agarose in 1% RPMI [42]. After 5 hours of treatment with fibronectin, 4×10^4^ MCF7 cells in 500 µL complete RPMI medium were plated onto multi-48-well containing a bottom layer of non-adherent 200 µL low melting agarose and kept at 37°C, 5% CO_2_ for 72 hours. After this period, the multicellular tumor spheroids (MTS) were transferred into new 48-well plates embedded on 400 µL 2.5 mg/ml type I collagen solution (BD Biosciences) in the presence or absence of 20 µg/mL fibronectin (Sigma) and incubated in a 37°C oven for 1 h. After this time 500 µL of complete medium was added over the joint. After seven days of submersion, the process of invasion into 3D collagen layers was observed and monitored under an inverted microscope. As a positive control for spheroid formation, the invasive cell line MDA-MB-435 that highly expresses MMP-2 was used with the same experimental conditions.

### Cloning and bisulfite sequencing of the CpG Island of the *MMP2* gene

Approximately 1 µg of DNA extracted with All-Prep kit (Qiagen), was used for bisulfite treatment with the Epitec reagent (Qiagen) according to the manufacturer’s specifications. The CpG island of the *MMP2* gene was amplified using the primers described previously [12], and the products were purified by 1% agarose gel electrophoresis using the Quick Extraction Protocols Kit (Qiagen). The purified products were then cloned into the pGEMT Easy vector (Promega) and electroporated into the *Escherichia coli* strain DH10B. Recombinant clones were selected by α-complementation. Plasmid DNA was extracted from at least eight clones using the QiaPrep kit (Qiagen). Approximately 300 to 500 ng of DNA were sequenced using the BigDye terminator kit on an XL Genetic Analyzer sequencer according to the manufacturer’s instructions (Applied Biosystems).

### Chromatin immunoprecipitation (ChIP)

MCF7 mock, FN-treated and recultured cells were used in this assay. After the cultures, the medium was aspirated and 1% formaldehyde in 10 mL of PBS was added. After 10 min of incubation at 37°C, the reaction was stopped with 2.5 M glycine. Approximately 2.5 ml of scraping buffer (PBS containing 1x protease inhibitor cocktail) was added to the plate, and cells were scraped from the plastic surface. The cells were centrifuged at 800×*g* at 4°C for 10 min and the pellet cells were lysed by lysis buffer supplied by MAGnify ChIP System (Invitrogen). The lysates were sonicated with Covaris sonicator for 13 cycles of 60 sec (under the conditions Duty - 5% Intensity - 2 Cycles/Burst - 200) and centrifuged at 14,000×*g* for 10 min at 4°C. Then, the supernatant was divided into 4 aliquots and immunoprecipitated with H3K27me3, H3K4me3, H3 and IgG (negative control) antibodies at a concentration of 2 µg/ml. The following steps were realized according to the MAGnify ChIP System (Invitrogen) manufacture’s instructions. The samples were quantified using Nanodrop 2000 and subjected to qPCR analysis.

For the ChIP qPCR assay, the following controls were tested at 400 nM: *MYOD1* and *GAPDH* (Qiagen). To compare all the samples, the endogenous *GAPDH* gene was chosen. We used 25 ng of immunoprecipitated DNA in qPCR with SYBR Green (Applied Biosystems) mix. B1 primers were used as described [12].

### Statistical analysis

A triplicate analysis was performed in all experiments, and values were analyzed by Student’s *t* Test *t* and *p*<0.05 was considered significant.

## Supporting Information

Figure S1 **Zymography analysis of MMP-2 from MCF7 cells.** M.M. molecular marker. PC, the breast tumor cell line MDA-MB-435 used as positive control. Mock; 5-Aza-treated and FN-treated. The bands correspond to the pro (72 kDa) and active (62 kDa) MMP-2 protein forms.
(TIF)

Figure S2 **Sequencing of the** ***MMP2*** **gene promoter in MDA-MB-436 cells.** Closed and open circles represent methylated or unmethylated CpGs, respectively. On the left the number represent the sequenced clones. The 49 analyzed CpGs in the *MMP2* promoter region of mock and FN-treated are shown. The global methylation percentage is also shown at right.
(TIF)

Figure S3 **Migration assay after fibronectin** **treatment in MCF7 cells.** Mock and FN-treated cells were submitted to a transwell haptotatic migration assay. The results consist of two independent experiments. No statistical differences were observed.
(TIF)

Figure S4 **Invasion assay after fibronectin assay in MCF7 cells.** Mock and FN-treated cells were transferred into a framework of agarose to form spheroids for 72 h. These spheroids were transferred to a collagen-containing matrix with or without fibronectin. The invasion into collagen was monitored for seven days. MDA-MB-435 cells were used as a highly invasive control.
(TIF)
