# Supplementary figures and images for: Fibronectin Affects Transient MMP2 Gene Expression through DNA Demethylation Changes in Non-Invasive Breast Cancer Cell Lines
Source: PLoS One. 2014 Sep 10;9(9):e105806. doi: 10.1371/journal.pone.0105806 (PMC4160184; doi:10.1371/journal.pone.0105806)

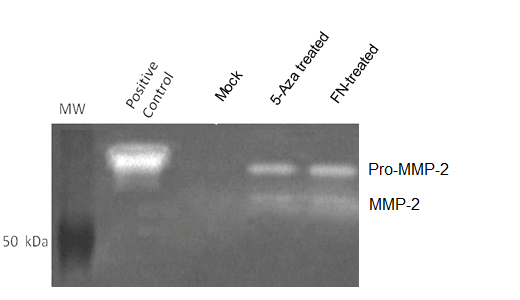

Supplement: Figure S1 — Zymography analysis of MMP-2 from MCF7 cells. M.M. molecular marker. PC, the breast tumor cell line MDA-MB-435 used as positive control. Mock; 5-Aza-treated and FN-treated. The bands correspond to the pro (72 kDa) and active (62 kDa) MMP-2 protein forms. (TIF) [file pone.0105806.s001.tif]

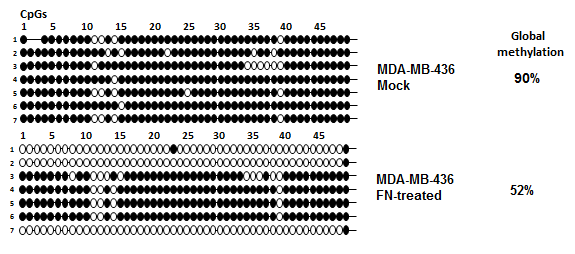

Supplement: Figure S2 — Sequencing of the MMP2 gene promoter in MDA-MB-436 cells. Closed and open circles represent methylated or unmethylated CpGs, respectively. On the left the number represent the sequenced clones. The 49 analyzed CpGs in the MMP2 promoter region of mock and FN-treated are shown. The global methylation percentage is also shown at right. (TIF) [file pone.0105806.s002.tif]

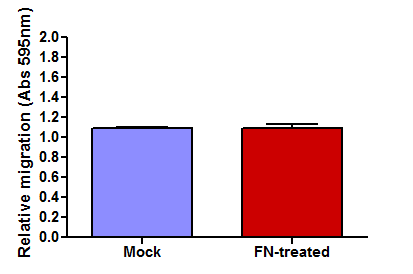

Supplement: Figure S3 — Migration assay after fibronectin treatment in MCF7 cells. Mock and FN-treated cells were submitted to a transwell haptotatic migration assay. The results consist of two independent experiments. No statistical differences were observed. (TIF) [file pone.0105806.s003.tif]

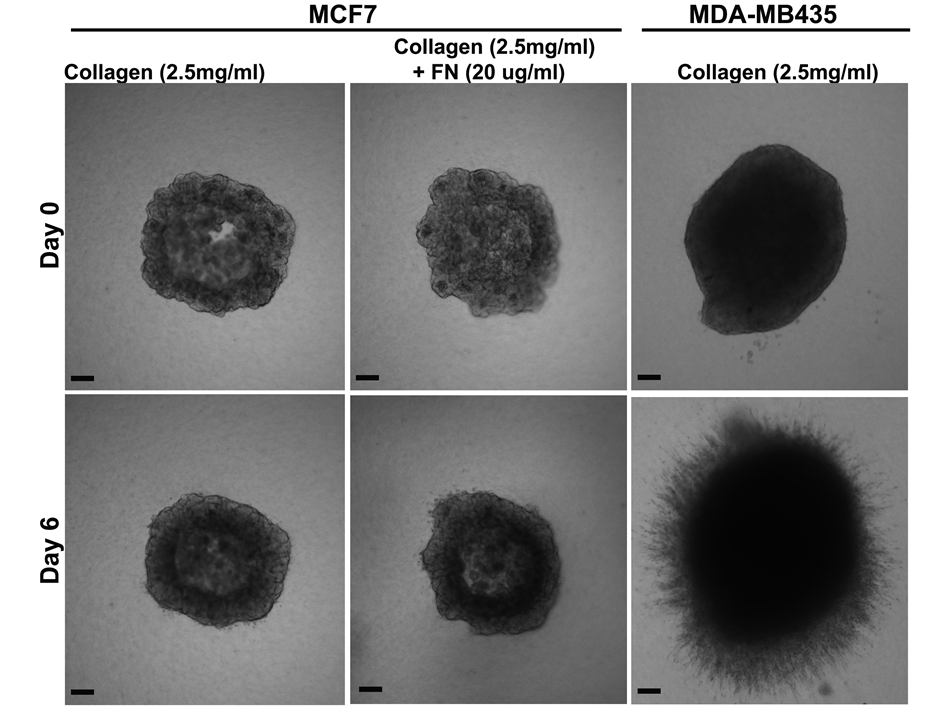

Supplement: Figure S4 — Invasion assay after fibronectin assay in MCF7 cells. Mock and FN-treated cells were transferred into a framework of agarose to form spheroids for 72 h. These spheroids were transferred to a collagen-containing matrix with or without fibronectin. The invasion into collagen was monitored for seven days. MDA-MB-435 cells were used as a highly invasive control. (TIF) [file pone.0105806.s004.tif]
